# Analysis of EGFR signaling pathway in nasopharyngeal carcinoma cells by quantitative phosphoproteomics

**DOI:** 10.1186/1477-5956-9-35

**Published:** 2011-06-28

**Authors:** Lin Ruan, Xin-Hui Li, Xun-Xun Wan, Hong Yi, Cui Li, Mao-Yu Li, Peng-Fei Zhang, Gu-Qing Zeng, Jia-Quan Qu, Qiu-Yan He, Jian-Huang Li, Yu Chen, Zhu-Chu Chen, Zhi-Qiang Xiao

**Affiliations:** 1 Key Laboratory of Cancer Proteomics of Chinese Ministry of Health, Xiangya Hospital, Central South University, Changsha 410008, China; 2 Key Laboratory of Allergy and Clinical Immunology, Department of Allergy, The Second Affiliated Hospital of Guangzhou Medical University, Guangzhou 510260, China; 3 Department of Biochemistry and Molecular Biology, College of Medicine, Hunan Normal University, Changsha 410006, China

## Abstract

**Background:**

The epidermal growth factor receptor (EGFR) is usually overexpressed in nasopharyngeal carcinoma (NPC) and is associated with pathogenesis of NPC. However, the downstream signaling proteins of EGFR in NPC have not yet been completely understood at the system level. The aim of this study was identify novel downstream proteins of EGFR signaling pathway in NPC cells.

**Results:**

We analyzed EGFR-regulated phosphoproteome in NPC CNE2 cells using 2D-DIGE and mass spectrometry analysis after phosphoprotein enrichment. As a result, 33 nonredundant phosphoproteins including five known EGFR-regulated proteins and twenty-eight novel EGFR-regulated proteins in CNE2 were identified, three differential phosphoproteins were selectively validated, and two differential phosphoproteins (GSTP1 and GRB2) were showed interacted with phospho-EGFR. Bioinformatics analysis showed that 32 of 33 identified proteins contain phosphorylation modification sites, and 17 identified proteins are signaling proteins. GSTP1, one of the EGFR-regulated proteins, associated with chemoresistance was analyzed. The results showed that GSTP1 could contribute to paclitaxel resistance in EGF-stimulated CNE2 cells. Furthermore, an EGFR signaling network based on the identified EGFR-regulated phosphoproteins were constructed using Pathway Studio 5.0 software, which includes canonical and novel EGFR-regulated proteins and implicates the possible biological roles for those proteins.

**Conclusion:**

The data not only can extend our knowledge of canonical EGFR signaling, but also will be useful to understand the molecular mechanisms of EGFR in NPC pathogenesis and search therapeutic targets for NPC.

## Background

Nasopharyngeal carcinoma (NPC) is one of the most common malignant tumors in Southern China [1]. Although NPC is a relatively radiosensitive disease, some of the NPC patients present local recurrences and distant metastases after radiotherapy due to radioresistance and the majority of these patients surrender recurrence and metastasis within 1.5 year after treatment [2]. Hence, development of a specific targeted therapy for NPC is urgent for improving the patient survival and prognosis. Uncovering signaling pathway involved in NPC cancer biology will provide important information on targeted therapy for this disease.

Overexpression of epidermal growth factor receptor (EGFR) is common in NPC [3-5], and most NPC cell lines and about 85% of the Chinese patients with NPC have moderate to strong expression of EGFR [6,7]. Moreover, overexpression of EGFR in primary tumors was associated with tumor metastasis, recurrence, and poor survival in patients with NPC [7,8]. Recent data have proposed EGFR as a new target for NPC therapy [9,10]. These studies suggest that EGFR plays a crucial role in the development and progression of NPC. EGFR is one of the most studied receptor tyrosine kinases. The natural ligands EGF and TGF-α bind to the extracellular domain of EGFR, and activate the receptor and its downstream signal proteins, ultimately causing activation or modulation of various cellular processes [11]. About 200 targets of EGFR signaling pathway have been reported [12], and 177 molecules involved in EGFR signaling pathway are listed in the Human Protein Reference Database http://www.hprd.org, but the downstream signaling proteins of EGFR in NPC have not yet been completely understood at the system level.

Signaling transduction is regulated by the phosphorylation and dephosphorylation of proteins. Phosphoproteomics has an advantage for investigating cellular signaling pathways by simultaneously identifying a number of phosphoproteins at one experiment, but it also has a technical challenge because of the low abundance of phosphoproteins in cells. Therefore, enrichment of phosphoproteins is necessary before starting a phophoproteomic analysis to increase the sensitivity of identifying phosphoproteins. Two-dimensional difference gel electrophoresis (2D-DIGE) is a quantitative proteomics approach with great sensitivity and accuracy of quantitation compared to a conventional 2-DE. Using the 2D-DIGE, different samples prelabeled with mass- and charge-matched fluorescent cyanine dyes are co-separated in the same 2D gel, and an internal standard is used in every gel, overcoming the problem of intergel variation in classical 2-DE. Therefore, 2D-DIGE is able to efficiently provide accurate and reproducible differential expression values for proteins in two or more biological samples [13,14].

To identify EGFR signaling proteins in NPC cells, in this study quantitative phosphoproteomics based on phosphate metal affinity chromatography-enriched phosphorproteins, 2D-DIGE and mass spectrometry analysis was applied to identify phosphoproteins after EGFR activation in NPC cells. We identified 33 EGFR-regulated phosphoproteins, and constructed an EGFR signaling network based on the identified phosphoproteins in NPC cells. The functional validation showed that GSTP1, one of the EGFR-regulated proteins, is involved in paclitaxel resistance in EGF-stimulated CNE2 cells. The data will provide insights into our understanding of EGFR signaling pathway and may have implications on target-directed therapeutics for NPC.

## Methods

### Cell culture and EGF treatment

NPC cell line CNE2 cells were cultured to 60-70% confluency in DMEM medium supplemented with 10% fetal bovine serum (Invitrogen) at 37°C, serum-starved for 24 h, and then were stimulated with 30 ng/mL EGF (Sigma) or mock-treated as a control. In EGFR blocking experiments, cells were pretreated with 1 μm EGFR tyrosine kinase inhibitor PD153035 (Calbiochem), and followed by incubation with EGF.

### Phosphoprotein enrichment

A phosphoprotein purification kit (BD Biosciences) was applied to enrich phosphoproteins from EGF-stimulated or unstimulated CNE2 cells according to the manufacturer's instructions. To validate the efficacy of phosphoprotein enrichment, 40 μg of proteins from total cellular lysate, elution fraction containing the highly-concentrated and purified phosphoproteins, and flow-through fraction were separated by 6% SDS-PAGE, followed by Western blotting using anti-phosphotyrosine antibody (4G10, Upstate). The concentration of the phosphoproteins was determined using a 2-D Quantification Kit (Amersham Biosciences).

### Protein labeling

Phosphoproteins from the elution fractions were precipitated using chloroform-methanol as described by Wessel and Flugge [15], resolubilized in 2D-DIGE sample buffer (30 mM Tris, 7 M urea, 2 M thiourea, 4% CHAPS, pH 8.5), and adjusted to pH 8.5. Equal amount phosphoproteins from six samples (three biological repeats) were pooled together as the internal standard. Three EGF-stimulated samples and three EGF-unstimulated samples were randomly labeled with Cy3 or Cy5, while internal standards were labeled with Cy2, using 200 pmol fluorochrome/25 μg protein (Amersham Biosciences). Labeling reactions were performed on ice in the dark for 30 min, and then quenched by the addition of 1 μL 10 mM lysine (Sigma) for 10 min.

### 2D-DIGE

Cy3- and Cy5-labelled samples (25 μg) from each pair of EGF-treated and untreated cells were combined before mixing with 25 μg Cy2-labelled internal standards. An equal volume of 2 × sample buffer (8 M urea, 130 mM DTT) was added to the sample and the total volume was made up to 450 μL with rehydration buffer (8 M urea, 4% CHAPS, 1% Biolyte, pH 4-7, 13 mM DTT). The samples were applied to IPG strips [pH 4-7; nonlinear (NL), 24 cm] and focused on an IPGphor (Amersham Biosciences). The focused IPG strips were equilibrated, and then were transferred to the tops of 12.5% polyacrylamide gels and run for about 7 h, using low-fluorescence glass plates on an Ettan DALT II system (Amersham Biosciences). All electrophoresis procedures were performed in the dark. The biological triplicate EGF-stimulated and unstimulated cells and the internal standard were run on three gels as analytic gels. In addition, we performed another strip in parallel as a preparative gel for spot pickings, as described above, except that the IPG strip was loaded with 1000 μg proteins and the gel was stained with Coomassie brilliant blue. After SDS-PAGE, the three analytic gels were scanned on a Typhoon 9410 scanner (GE Healthcare) at appropriate excitation/emission wavelengths specific for Cy2 (488/520 nm), Cy3 (532/580 nm) and Cy5 (633/670 nm), to generate nine protein spot maps.

### Image analysis

Images were cropped using ImageQuant TL 2005 software (GE Healthcare) from 2D-DIGE gels, and analyzed using DeCyder 6.5 software (GE Healthcare) according to the manufacturer's recommendations. The DeCyder differential in-gel analysis (DIA) module was used for pairwise comparisons of each EGF-stimulated and unstimulated cell sample to the internal standard in each gel. The DeCyder biological variation analysis (BVA) module was then used to simultaneously match all nine protein-spot maps and, using the Cy3: Cy2 and Cy5: Cy2 DIA ratios, to calculate average abundance changes and paired Student's *t *-test *p *values for the variance of these ratios for each protein pair across all samples. The differential protein spots (ratio ≥ 1.5, *p *≤ 0.05) that altered consistently in all nine protein-spot maps were selected for identification.

### Protein identification by MS

All the differential phosphoprotein spots were excised from stained preparative gels using punch, destained, and in-gel trypsin digestion was performed as previously described by us [16]. Briefly, the gel spots were destained with 100 mM NH4HCO3 in 50% acetonitrile, dried in a vacuum centrifuge, and incubated in the digestion solution (40 mM NH4HCO3, 9% acetonitrile, and 20 μg/mL proteomics grade trypsin) at 37°C for 14-16 h. The resulted peptides were extracted with 50% acetonitrile/2.5% TFA, purified with ZipTip C18 column (Millipore) and mixed with CCA matrix solution[4-hydroxy-*α*-cyanocinnamic acid (HCCA; Sigma) in 30% ACN/0.1% TFA] followed by analysis with Voyager System DE-STR 4307 MALDI-TOF Mass Spectrometer (ABI) to obtain the peptide mass fingerprint (PMF). The standard peptide mixture was analyzed at the same time to correct the machine. The parameters of MALDI-TOF were set up as follows: positive ionreflector mode, accelerating voltage 20 kV, grid voltage 64.5%, mirror voltage ratio 1.12, N2 laser wavelength 337 nm, pulse width 3 ns, the number of laser shots 50, acquisition mass range 500-3000 Da, delay 100 ns, and vacuum degree 4 × 10^-7 ^Torr.

In peptide mass fingerprint map database searching, Mascot Distiller was used to obtain the monoisotopic peak list from the raw mass spectrometry files. Peptide matching and protein searches against the Swiss-Prot database were performed using the Mascot search engine http://www.matrixscience.com/ with a mass tolerance of ± 50 p.p.m. Protein scores of ≥ 56 (threshold) indicate identity or extensive homology (*P *< 0.05) and were considered significant.

### Bioinformatics analysis

To do phosphorylation site prediction of the identified proteins, we used on-line PhosphoSitePlus™ system biology resource http://www.phosphosite.org/ and the Phospho.ELM database http://phospho.elm.eu.org/index.html for predicting the presence of the phosphorylation modification sites [17], and PubMed database searching http://www.ncbi.nlm.nih.gov for comparing with the phosphorylated proteins reported in the literature. In addition, KEGG pathway analysis of the identified proteins was done in DAVID bioinformatics resources [18,19].

### Validation of EGFR signaling phosphoproteins by IP-Western blotting

Cells were lysed in the lysis buffer containing 150 mM NaCl, 50 mM Tris-HCl (pH 7.4), 1 mM EDTA, 1% Ttiton X-100, 1% NP-40 supplemented with phosphatase inhibitor cocktail 1 and 2 (Sigma-Aldrich) at 4°C, and subsequently centrifuged at 12000 g for 30 min at 4°C. Total cellular proteins were immunoprecipitated with protein G Sepharose (Amersham Biosciences), and anti-target protein antibody overnight at 4°C. Immunocomplexes were used for Western blotting. Briefly, proteins were separated by 7% SDS-PAGE, and transferred to a PVDF membrane. Blots were blocked with 3% BSA for 1 h at room temperature, and then incubated with primary antibody, followed by incubation with HRP-conjugated secondary antibodies for 1 h at room temperature. The signal was visualized using ECL detection reagent.

### Transient transfection

The cells were transfected with GSTP1 siRNA or control siRNA (Santa Cruz Biotechnology) according to the siRNA transfection protocol provided by the manufacturer. Briefly, the day before transfection, CNE2 cells were plated into 6-well plates at the density of 10^5 ^cells/mL in DMEM medium containing 10% FBS (Invitrogen). When the cells were 60-80% confluent, they were transfected with 10 nmol/L of GSTP1 siRNA or control siRNA in serum-free DMEM medium using Lipofectamine 2000 reagent (Invitrogen). 4 h after the beginning of the transfection, the medium was replaced with DMEM medium containing 10% FCS, and continued to culture the cells for additional 44 h, and then GSTP1 expression level was determined by Western blotting.

### Flow cytometry analysis of apoptotic cells after treatment of paclitaxel and EGF

At the end of the transfection, the cells were incubated with 30 nM paclitaxel and 30 ng/mL EGF for 48 h, and cell apoptosis was examined by flow cytometry as previously described by us [20]. Briefly, cells were harvested, fixed with ice-cold 70% ethanol in PBS at -20°C for 1 h and then centrifuged at 1 500 rpm for 5 min. The pellets were incubated with 0.5% Triton X-100 (Sigma) and 0.05% RNase (Sigma) in 1 mL PBS at 37°C for 30 min, and then centrifuged at 1 500 rpm for 5 min. The cell pellets were incubated with 40 μg/mL propidium iodide (Sigma) in 1 mL PBS at room temperature for 30 min. Samples were immediately analyzed by a FACScan flow cytometry (Becton Dickinson). Apoptosis was evaluated based on the proportion of sub-G1 hypodiploid cells. Three independent experiments were done.

### Analysis of cell viability by MTT after treatment of paclitaxel and EGF

At the end of the transfection, the cells were incubated with the different concentrations of paclitaxel and 30 ng/mL EGF for 48 h, and cell viability was examined using MTT assay as previously described by us [20]. Briefly, 20 μl of 5 mg/mL MTT (Sigma) was added to each well, and the medium was removed after 4 h of incubation. 150 μL DMSO (Sigma) was added to each well for 10 min at room temperature. The absorbance of each well was read with a Bio-Tek Instruments EL310 Microplate Autoreader at 490 nm. Three independent experiments were done.

### Network modeling

To construct EGFR signaling network (biological interaction network of the proteins) of identified phosphoproteins, functional and pathway analyses were performed using Pathway Studio 5.0 software, a tool for the description of networks and signaling pathways [21].

## Results

### Enrichment of phosphoproteins in EGF-stimulated and unstimulated NPC cells

**Figure 1:**
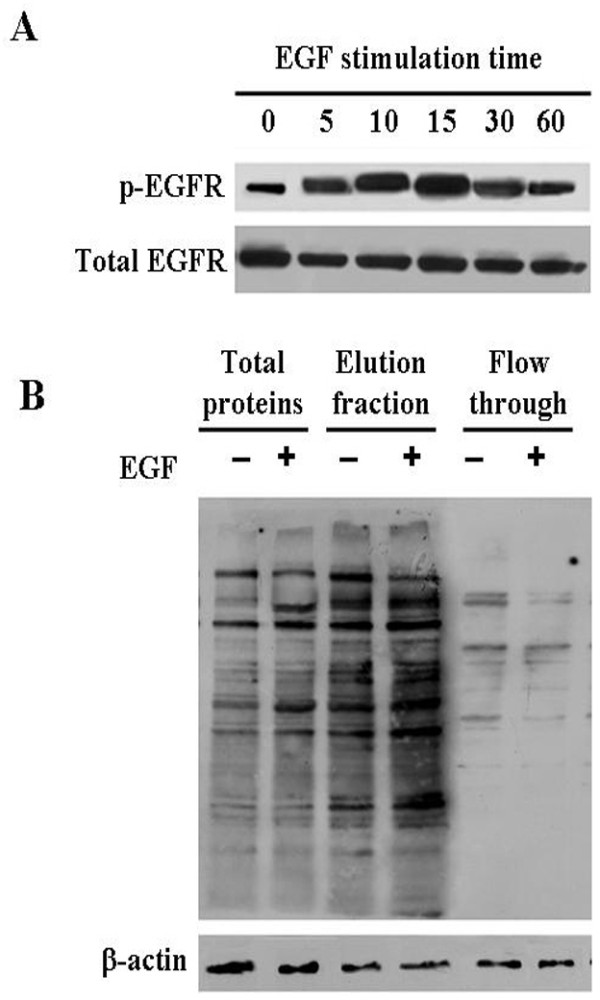
**Enrichment of phosphoproteins in EGF-stimulated and unstimulated NPC cells**. A, Detection of EGFR activation by Western blotting. CNE-2 cells were stimulated with 30 ng/mL EGF for different times, and Western blotting was performed to detect levels of phosphorylated EGFR in total cellular proteins. Total EGFR serves as a loading control. B, Enrichment of phosphoproteins. Phosphoprotein enrichment kit was used to enrich phosphoproteins from total cellular proteins of EGF-stimulated and unstimulated CNE2 cells, and Western blotting analysis of phosphoproteins using anti-phosphotyrosine antibody(4G10) in the total cellular protein, elution fraction and flow through fraction. β-actin serves as a loading control.

A commercial phosphoprotein enrichment kit based on phosphate metal affinity chromatography was used to enrich phosphoproteins from EGF-stimulated and unstimulated NPC CNE2 cells. Typically, the elution fraction contains highly-concentrated and purified phosphoproteins. As shown in Figure 1A, levels of phosphorylated EGFR in CNE2 reached the high peak 15 min after 30 ng/mL EGF-stimulated cells. Then the total proteins of cells treated by ng/mL EGF for 15 min were used to enrich phosphoproteins. As shown in Figure 1B, the elution fractions contained more phosphoproteins compared with the total cellular proteins and flow-through fractions, indicating that the elution fractions can be used to identify EGFR-regulated phosphoproteins.

### Identification of differential phosphoproteins in EGF-stimulated and unstimulated NPC cells by 2D-DIGE and MS

**Figure 2:**
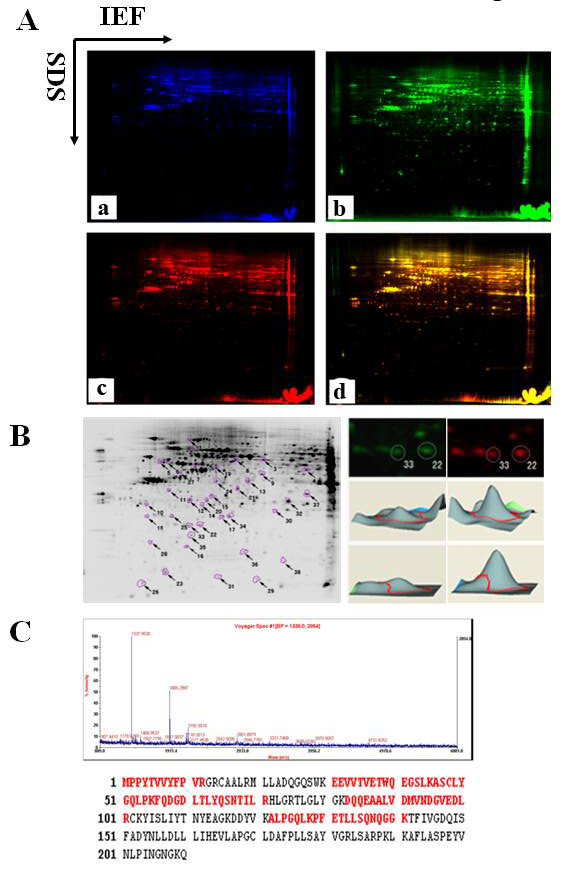
**Comparative phosphoproteomics analysis of EGF-stimulated and unstimulated NPC cells by 2D-DIGE and MS**. A, Representative 2D-DIGE gel images. Internal standard labeled with Cy2 (a, blue), phosphoproteins from EGF-unstimulated cells labeled with Cy3 (b, green), phosphoproteins from EGF-stimulated cells labeled with Cy5 (c, red), and overlaying Cy3 and Cy5 images (d, yellow). B, (Left) the 38 differential phosphoprotein spots detected by Decyder software; (Right) a close-up of the region of 2D-DIGE gel images and a 3D simulation of spots 22 and 33. C, MALDI-TOF-MS analysis of differential protein spot 22. Mass spectrum of spot 22 identified as GSTP1 according to the matched peaks is shown (top). Protein sequence of GSTP1 is shown, and matched peptides are labeled in red letters (bottom).

2D-DIGE and MS analysis were performed to identify differential phosphoproteins in EGF-stimulated and unstimulated (control) CNE2 cells. As shown in Figure 2A, phosphoproteins were labeled with either Cy3 (EGF-stimulated cells) or Cy5 (control) fluorescent dyes, while internal standards were labeled with Cy2. The interchangeable use of either Cy3 or Cy5 for each experiment has already been established. After 2D-DIGE, the Cy2, Cy3 and Cy5 images were scanned and analyzed using DeCyder software. 38 protein spots were differentially expressed in all nine protein-spot maps (Figure 2B left); 33 nonredundant proteins were identified by MS (Table 1); among them, five proteins are known EGFR-regulated proteins (KRT8, hnRNPK, KRT18, GRB2, Stathmin), and the other twenty-eight proteins have not been reported as EGFR-regulated proteins. A close-up of the region of 2D-DIGE gel images and a three-dimensional (3D) simulation of spots 22 and 33 significantly up-regulated in EGF-stimulated cells compared with control are shown in Figure 2B (right). MALDI-TOF-MS analysis and database matching identified spot 22 as Glutathione S-transferase P 1(GSTP1) with high sequence coverage and mass accuracy (Figure 2C).

**Table 1:** Identification of differential phosphoproteins in EGF-stimulated and unstimulated NPC CNE2 cells by 2-D DIGE and MS

**No**.	Protein name	Gene name	Uniprot accession	Mw (Da)	*p*I	Number of Matched	Sequence coverage	Score	Ratio (experiment/control)		Phosphorylation sites
										
						peptides	(%)		Ratio	*t *test	
1	Translation endoplasmic reticulam ATPase (TERA)	VCP	P55072	89950	5.14	16	31%	148	1.75	0.0082	Yes
2	Heat shock cognate 71kDa protein	HSP7C	P11142	71082	5.37	13	38%	118	-1.51	0.018	Yes
3	Keratin,type Ⅱ cytoskeletal 8	KRT8	P05787	53671	5.52	18	43%	139	3.47	0.0035	Yes
4	BAG family molecular chaperone regulator 5	BAG5	Q9UL15	51738	5.76	4	15%	56	-2.51	0.0001	No
5	TUBB protein	TUBB	P07437	50095	4.78	25	62%	278	3.12	0.0009	Yes
6	Vimentin	VIM	P08670	53676	5.06	12	30%	102	1.99	0.0056	Yes
7	Heterogeneous nuclear ribonucleoprotein K	hnRNPK	P61978	51230	5.39	8	31%	86	2.89	0.0005	Yes
8	60KD Heat shock protein	HSP60	P10809	61187	5.70	12	29%	109	2.20	0.008	Yes
9	Keratin,type Ⅱ cytoskeletal 8	KRT8	P05787	53671	5.52	6	36%	85	1.63	0.032	Yes
10	Proliferating cell nuclear antigen	PCNA	P12004	29092	4.57	9	52%	93	2.88	0.0001	Yes
11	F-actin-capping protein subunit beta	CAPZB	P47756	31616	5.36	5	26%	56	2.23	0.009	Yes
12	laminin receptor 1	RSSA	P08865	32947	4.79	7	38%	76	1.76	0.0056	Yes
13	Creatine kinase B-type	CKB	P12277	42902	5.34	7	38%	100	1.99	0.0021	Yes
14	Emerin	EMD	P50402	28994	5.29	6	21%	77	1.71	0.0014	Yes
15	Heterogeneous nuclear ribonucleoproteins C1/C2	hnRNP C1/C2	P07910	33707	4.95	5	18%	68	2.98	0.0008	Yes
16	ATP synthase D chain mitochondrial	ATP5H	O75947	18537	5.21	5	37%	57	2.67	0.005	Yes
17	Prohibitin	PHB	P35232	29843	5.57	7	37%	120	-2.54	0.031	Yes
18	unidentified								1.79	0.002	N/A
19	Elongation factor 1- delta	EEF1D	P29692	32.217	4.9	4	25%	72	1.81	0.026	Yes
20	Anamorsin	CIAPIN1	Q6FI81	34141	5.44	9	39%	139	2.90	0.0001	Yes
21	Annexin A3	ANXA3	P12429	36524	5.63	7	27%	83	2.83	0.0054	Yes
22	Glutathione S-transferase P1	GSTP1	P09211	23569	5.43	6	34%	97	3.11	0.0013	Yes
23	unidentified								1.64	0.026	N/A
24	Keratin,typeⅠcytoskeletal 18	KRT18	P05783	48029	5.34	13	36%	141	-2.86	0.029	Yes
25	Ran-specific GTPase- activating protein	RANBP1	P43487	23310	5.71	6	32%	98	1.89	0.034	Yes
26	unidentified								1.68	0.007	N/A
27	Tropomodulin-3	TMOD3	Q9NYL9	39.595	5.08	8	34%	98	2.61	0.003	Yes
28	Myosin light polypeptide 6	MYL6	P60660	17090	4.56	6	50%	70	2.80	0.0019	Yes
29	Nucleoside diphosphate kinase A (NDKA)	NME1	P15531	17309	5.83	6	48%	105	-1.56	0.0023	Yes
30	Heat shock protein beta-1	HSP27	P04792	22826	5.98	7	47%	84	2.65	0.0001	Yes
31	unidentified								-2.72	0.038	N/A
32	Growth factor receptor-bound protein 2	GRB2	P62993	25,206	5.89	7	39%	75	2.60	0.00028	Yes
33	Glutathione S-transferase P1	GSTP1	P09211	23569	5.43	6	43%	91	3.99	3.2E-06	Yes
34	Peroxiredoxin-2	PRDX2	P32119	22049	5.66	6	54%	98	-1.69	0.042	Yes
35	c-Myc-responsive protein	RCL	O43598	19211	4.97	4	25%	67	2.25	0.009	Yes
36	Protein DJ-1	PARK7	Q99497	19.891	6.33	5	30%	65	2.99	0.0036	Yes
37	3-Hydroxyisobutyrate dehydrogenase mitochondrial precursor (3HIDH)	HIBADH	P31937	35705	8.38	4	22%	95	-2.55	0.0051	Yes
38	Stathmin	STMN1	P16949	17171	5.77	6	19%	95	2.57	0.0023	Yes

### Bioinformatics analysis of the identified proteins

Phosphorylation modification sites of 33 identified proteins were analyzed with two online resources (PhosphoSitePlusTM system biology resource, and the Phospho.ELM database) to confirm the identified proteins being phosphoproteins. The results showed that 32 of 33 identified proteins contain phosphorylation modification sites except BAG5 (Table 1). In addition, KEGG pathway analysis showed that 17 identified proteins (HSP7C, KRT8, TUBB, VIM, HSP60, PCNA, RSSA, CKB, ATP5H, STMN1, GSTP1, KRT18, NME1, HSP27, GRB2, PARK7, and 3HIDH) are signaling proteins involved in MAPK, JAK/STAT and VEGF pathways, etc.. Taken together, these results support that the proteins identified by phophoproteomics are phosphoproteins.

### Validation of identified phosphoproteins

**Figure 3:**
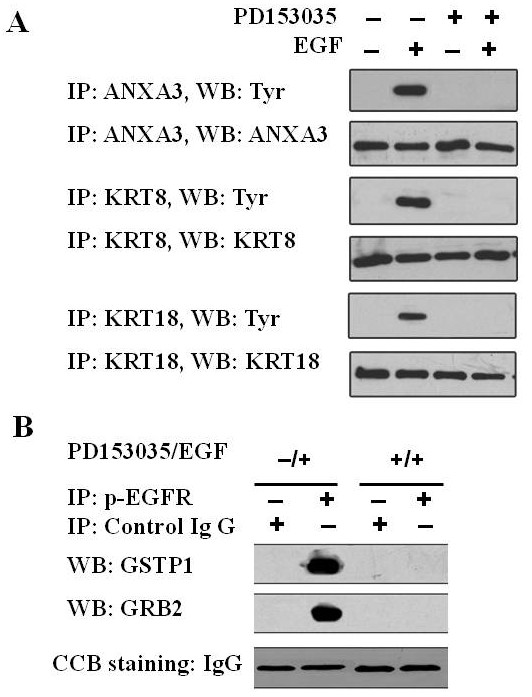
**Validation of EGFR-regulated phosphoproteins by IP-Western blotting**. A, IP-Western blotting analysis showing that EGF induces phosphorylation of three identified proteins (ANXA3, KRT8 and KRT18). Try, anti-phosphotyrosine antibody B, IP-Western blotting analysis showing that phospho-EGFR interacts with GSTP1 and GRB2. CBB gel staining serves as loading control (IgG heavy chain).

To confirm the results of phosphoproteomics, we detected the phosphorylated levels of three identified proteins (ANXA3, KRT8, and KRT18) by IP-Western blotting. Following immunoprecipitation (IP) of ANXA3, KRT8, and KRT18 from total cellular proteins, immunocomplexes were analyzed by Western blotting using anti-phosphotyrosine antibody. As shown in Figure 3A, the levels of phosphotyrosine of ANXA3, KRT8, and KRT18 were significantly higher in the 30 ng/mL EGF-stimulated CNE2 cells than in EGF-unstimulated CNE2 cells, and tyrosine phosphorylation of ANXA3, KRT8, and KRT18 could be blocked by the pretreatment of the cells with 1 μm EGFR inhibitor PD153035. The results indicate that EGFR activation can induce phosphorylation of ANXA3, KRT8, and KRT18, which is consistent with results of phosphoproteomics.

### Interaction of identified proteins with phospho-EGFR

IP-Western blotting were performed detect whether activated (phosphorylated) EGFR interacted with the two identified phosphoproteins (GSTP1 and GRB2) in CNE2 cells. As shown in Figure 3B, GSTP1 and GRB2 could be detected in the immunoprecipitation complex of phospho-EGFR antibody in 30 ng/mL EGF-stimulated CNE2 cells, and could not be detected by the pretreatment of the cells with 1 μm EGFR inhibitor PD153035, which indicates that GSTP1 and GRB2 can interact with phospho-EGFR, are downstream targets of EGFR signaling pathway.

### Association of EGFR-regulated GSTP1 with chemoresistance

**Figure 4:**
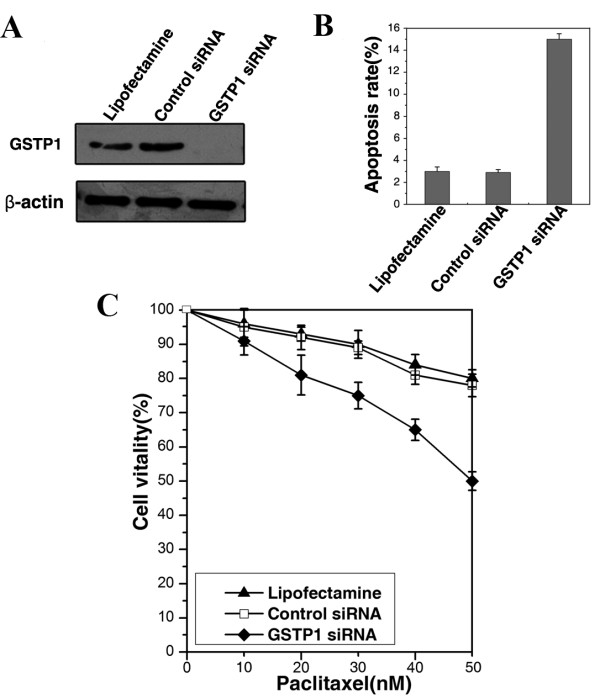
**The effect of GSTP1 siRNA transfection on paclitaxel sensitivity in EGF-stimulated CNE2 cells**. A, Western blotting analysis showed that transfection of CNE2 cells with GSTP1 siRNA knocked down GSTP1 expression, whereas GSTP1 expression was not significantly suppressed by control siRNA. B, Flow cytometric analysis showed that GSTP1 siRNA transfection could significantly increase cell apoptosis in CNE2 cells incubated with paclitaxel and EGF. C, MTT assay showed that GSTP1 siRNA transfection could significantly decrease the cell viability of CNE2 cells incubated with paclitaxel and EGF. Lipofectamine, cells treated with lipofectmine only.

To study the functional role of EGFR-regulated GSTP1 in CNE2 cells, CNE2 cells were transfected with GSTP1 siRNA. As shown in Figure 4A, GSTP1 siRNA transfection knocked down GSTP1 expression in CNE2 cells, whereas GSTP1 expression was not significantly suppressed by control siRNA. We next evaluated the effects of GSTP1 siRNA transfection on the paclitaxel sensitivity in EGF-stimulated CNE2 cells. CNE2 cells transfected with GSTP1 siRNA or control siRNA were incubated with paclitaxel and EGF for additional 48 h. And then the cell apoptosis and cell viability were examined using flow cytometry and MTT assay, respectively. As Figure 4B and 4C shown, compared with control siRNA transfection, GSTP1 siRNA transfection could enhance EGF-stimulated CNE2 cells to paclitaxel sensitivity, with the significant increase of apoptotic cells and decrease of cell viability, which demonstrates that EGFR-regulated GSTP1 is involved in chemoresistance in CNE2 cells.

### Construction of EGFR signaling network in NPC cells

**Figure 5:**
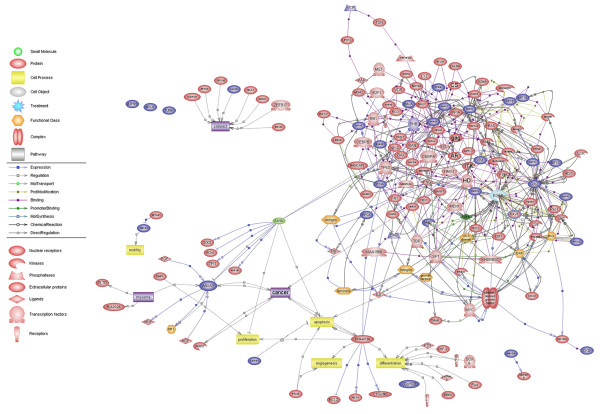
**Signaling network of EGFR-regulated phosphoproteins in NPC cells**. Proteins identified in Table 1 were imported into Pathway Studio, and an interaction map was generated with information from ResnetCore of Ariadne Genomics, and KEGG Pathway database. Legend of the interaction network is summarized on the right of the figure. Phosphoproteins, which are identified as phosphorylated targets of EGF signaling, were highlighted on the map (blue).

Based on the identified phosphoproteins, we constructed EGFR signaling network using Pathway Studio 5.0 software. The result showed that 85% (28/33) proteins could be networked (Figure 5). The proteins that could be networked were linked by various relationships such as protein binding, protein interactions, modifications including phosphorylation, and expression regulation. The biological interaction network has a biological significance beyond static phosphoproteome data, suggesting that the majority of the proteins identified in this study were integral part of the dynamic complex of EGFR signaling.

## Discussion

EGFR play an important role in development, progression and therapeutic resistance of NPC [6-8], but the role and mechanisms of EGFR in the NPC are not fully understood. Activation of EGFR initiates important cell signaling cascades such as PI3K/AKT/mTOR, JAK/STAT, and Ras/Raf/MAPK pathways [22]. About 200 targets of EGFR signaling pathway have been reported [12], and 177 molecules involved in EGFR signaling pathway are listed in the Human Protein Reference Database http://www.hprd.org, but EGFR signaling pathway in NPC still needs to be elucidated.

In the present study, we used quantitative phosphoproteomics to identify EGFR-regulated phosphoproteins to elucidate EGFR signaling pathway in NPC cells at the system level. 33 proteins were identified in enriched phosphoproteins from EGFR-activated NPC CNE2 cells, and three phosphoproteins were validated by IP-Western blotting. Among the identified proteins, five are known EGFR signaling proteins and twenty-eight are novel EGFR signaling proteins. To confirm the phosphoproteins identified by phosphoproteomics, online bioinformatics resources were used to predict phosphorylation sites of 33 identified proteins. The results showed that 32 proteins contain phosphorylation modification sites. KEGG pathway analysis also showed that 17 identified proteins are signaling proteins. Taken together, these results support that the proteins identified by phosphoproteomics are phosphoproteins. Interestingly, our result showed that two identified proteins (GSTP1 and GRB2) could interact with phospho-EGFR in EGF-stimulated CNE2 cells, further supporting that the identified phosphoproteins are EGFR signaling proteins.

To uncover the biological context of EGFR signaling proteins, we constructed a biological interaction network of the identified phosphoproteins, which has biological significance beyond static phosphoproteome data. Interestingly, 28 of 33 identified phosphoproteins could be networked. This strongly suggests that the majority of the phophoproteins identified in this study were integral part of the dynamic complex of EGFR signaling. The proteins that could be networked were linked by various relationships such as protein binding, protein interactions, modifications including phosphorylation, and expression regulation. This biological interaction network will be useful for formulating testable hypotheses to understand the function of novel phosphorylated targets of EGFR signaling pathway in NPC cells.

GSTP1, a major drug-metabolizing and stress response signaling protein, belongs to GST family member [23]. Overexpression of GSTP1 has been reported in various types of human tumors, including colon cancer [24], gastric cancer [25], esophageal cancer [26], and head and neck squamous carcinoma [27], and enhances human cancer cell chemoresistance [23,28]. Chen reported that 58% (83/143) primary NPC, 69.8% (30/43) recurrent NPC, and 65% (13/20) metastatic NPC tissues highly expressed GSTP1 [29]. Jayasurya reported that all 55 NPC tissues showed positive GSTP1 immunoreactivity, and a significant correlation was found between GSTP1 expression and regional nodal metastasis of NPC [30]. In tumors with EGFR aberrant activation, GSTP1 was phosphorylated and activated, leading to drug inactivation and drug resistance [31]. Our results showed that activation of EGFR induced GSTP1 phosphorylation and interaction with EGFR, and GSTP1 is an important downstream target of EGFR signaling network in NPC cells. Overexpression of EGFR is frequent in NPC cell lines and tissues [6,7], and is associated with chemoresistance [32]. To explore the effect of EGFR-regulated GSTP1 in EGFR-induced chemoresistance in NPC cells, we evaluated the effects of GSTP1 knockdown on the paclitaxel sensitivity in EGF-stimulated CNE2 cells, and found that knockdown of GSTP1 expression by siRNA could increase EGF-stimulated CNE2 cells to paclitaxel sensitivity. It was obvious that GSTP1 is involved in EGFR-mediated chemoresistance in NPC cells. Our findings suggest that in NPC therapy, the double targeting of EGFR and GSTP1 could, potentially, be more effective than the current strategy of targeting either protein individually.

## Conclusion

In this study, we identified 33 EGFR signaling proteins using quantitative phosphoproteomics, constructed an EGFR signaling network based the identified phosphoproteins in NPC cells, and validated that GSTP1, one of the EGFR-regulated proteins, is involved in chemoresistance in NPC cells. The data not only can extend our knowledge of canonical EGFR signaling, but also will be useful to understand the molecular mechanisms of EGFR in NPC pathogenesis and search therapeutic targets for NPC.

## Competing interests

The authors declare that they have no competing interests.

## Authors' contributions

ZQX designed the experiments and prepared the manuscript. LR, XHL and XXW performed most of the experiments. The other authors participated in the experiments. All authors read and approved the final manuscript.
